# Peripheral CD19^hi^ B cells exhibit activated phenotype and functionality in promoting IgG and IgM production in human autoimmune diseases

**DOI:** 10.1038/s41598-017-14089-2

**Published:** 2017-10-24

**Authors:** Zhicui Liu, Weihong Zeng, Xiangyang Huang, Shujun Wang, Jie Zheng, Meng Pan, Ying Wang

**Affiliations:** 1 Department of Dermatology, Ruijin Hospital, Shanghai Jiao Tong University School of Medicine, 200025 Shanghai, P. R. China; 2 Shanghai Institute of Immunology, Shanghai Jiao Tong University School of Medicine, 200025 Shanghai, P. R. China; 3 Institute of Embryo-Fetal Original Adult Disease Affiliated to Shanghai Jiao Tong University School of Medicine, the International Peace Maternity & Child Health Hospital, Shanghai Jiao Tong University School of Medicine, 200030 Shanghai, P. R. China; 4 Department of Rheumatology and Immunology, West China Medical School of Sichuan University, 610041 Chengdu, Sichuan P. R. China

## Abstract

Systemic Lupus Erythematosus (SLE) and pemphigus are two representative autoimmune diseases driven by pathogenic autoantibody systemically and organ-specifically, respectively. Given the involvement of antibody in the pathogenesis, B cells are inclined to differentiate and function in an abnormal activation model. Here we defined a unique CD19^hi^ B cell population existing in the periphery of SLE and pemphigus patients as well as in human tonsils. CD19^hi^ B cells could be induced *in vitro* after co-culturing fully activated CD4^+^ T cells with autologous B cells. They expressed high levels of HLA-DR, IgG, IgM and multiple ligands of costimulatory molecules with the capacity to produce extra IgG and IgM. Transcirptome assay revealed that genes involved in B-cell activation and differentiation were up-regulated in CD19^hi^ B cells. Antibody blockade experiments showed that the interactions between costimulatory molecules contributed to CD19^hi^ B-cell generation and IgG/IgM production. What is more, frequencies of peripheral CD19^hi^ B cells from SLE and pemphigus patients were correlated with serum total IgG and IgM, but not with autoantigen-specific antibodies and disease severity. Therefore, our investigation demonstrates that CD19^hi^ B cells might contain B cell precursors for terminal differentiation and contribute to total IgG/IgM production in human autoimmune diseases.

## Introduction

Systemic lupus erythematosus (SLE) is a systemic autoimmune disease which is characterized as multi-organ damages through the deposition of auto-antibodies and immune complex^1^, while pemphigus is an organ-specific autoimmune disease bearing suprabasal blisters in skin and mucous membranes caused by autoantibodies against intercellular adhesion structures of epidermal keratinocytes^2^. Although the initiation of SLE and pemphigus is not yet fully understood, abnormal activation of B cells is demonstrated to play central roles in the development and progression of both SLE and pemphigus with the presence of pathogenic autoantibodies in the periphery of the patients, such as anti-nuclear antibodies (ANA) in SLE^3^ and anti-desmoglein 3 (Dsg 3)/Dsg 1 autoantibodies in pemphigus^4^. Pathogenic dissection of autoantibody-driven autoimmune diseases, such as SLE and pemphigus, will thus be of great value to elucidate the mechanisms of human B cell activation as well as to identify the targets for the treatment of the diseases.

Recent progresses in B-cell activation and differentiation have drawn a picture of the complexity with multi-steps in the generation of long-lived plasma cells (PCs) and memory B cells in the follicles of germinal centers (GCs)^5^ as well as extra-follicular plasmablasts^5,6^. B cell activation is triggered by antigen recognition through B-cell antigen receptor (BCR) either directly or with the help of antigen presenting cells (APCs) in peripheral lymphoid organs, and is achieved by the activation of intracellular signaling pathways and subsequent target gene expression. The activated B cells migrate to B-T area of lymphoid organs where they undergo a limited expansion upon cognate interaction with antigen-primed T cells. A fraction of B cells differentiate into short-lived plasmablasts providing prompt responses to antigens, while others initiate the formation of GC in secondary follicles. The activated B cells interact with follicular helper T cells (Tfh)^7^ in GCs where they undergo somatic hyper-mutation (SHM) to generate BCR with higher affinity to antigens^5,8^, and class switch recombination (CSR) for subtypic immunoglobulin. B cells finally differentiate into long-lived PCs and memory B cells^9^. However, the complexity of how B-cell differentiation being linked to antibody generation in autoimmune diseases is unclear.

In fact, unlike the widely understanding of T cell subsets involved in human diseases, the clinical significance of B cell subsets or those at different differentiation stages is still very limited. Recently, regulatory B cells are reported to be involved in several antibody-driven autoimmune diseases, including SLE^10,11^ and pemphigus^12^. CD19^hi^ B cell is another subset that was firstly reported in patients with common variable immunodeficiency (CVID) as a potential biomarker for autoimmune cytopenia and splenomegaly^13^. Later on, this population was found to be expanded in SLE patients with an activation phenotype and extralymphatic homing property^14^. They are supposed to be the precursors of autoimmune PCs with poor clinical outcomes in SLE patients^15^. However, the generation and property of pathogenic CD19^hi^ B cells are not well defined yet.

We reported here the existence of CD19^hi^ B cell subset in the periphery of SLE and pemphigus patients as well as in human tonsils. They were induced under the help of activated CD4^+^ T cells *in vitro* with unique phenotype and functionality. Gene expression profiles were further investigated by using genome-wide microarrays. With strong correlation between peripheral CD19^hi^ B cells and total IgG/IgM levels in SLE and pemphigus patients, it is deduced that CD19^hi^ B cells might contain a distinct B cell subset contributing to abnormal IgG/IgM production in human autoimmune diseases.

## Results

### Presence of CD19^hi^ B cells in the periphery of SLE and pemphigus patients as well as in human tonsils with activated and memory-like phenotypes

**Figure 1:**
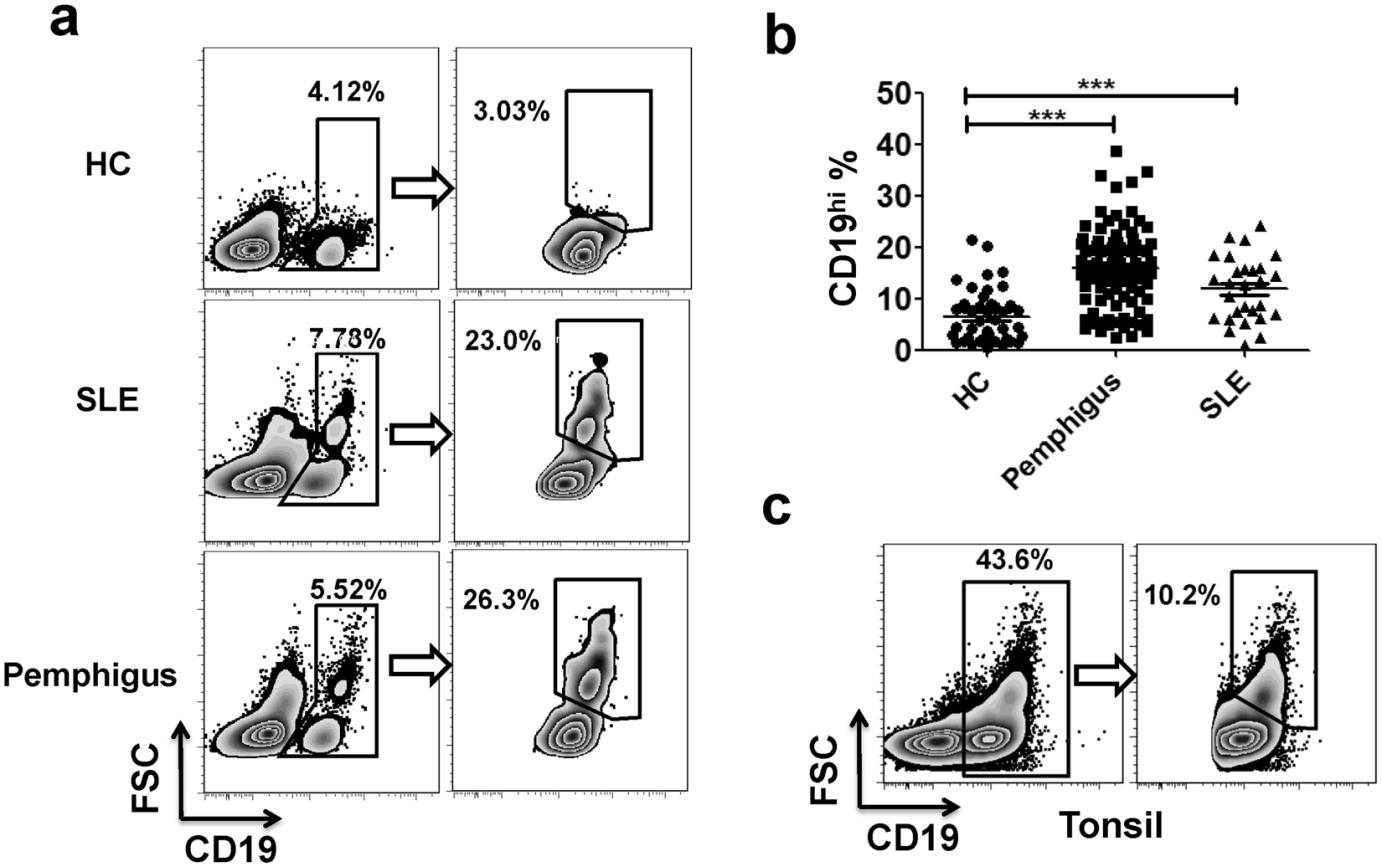
CD19^hi^ B cells exist in the periphery of SLE and pemphigus patients as well as in human tonsils. (**a)** Flow cytometric histograms of representative CD19^hi^ B cells in the periphery of HCs, SLE and pemphigus patients based on CD19 expression and cell size (FSC). **(b)** Comparison of CD19^hi^ B cells’ percentages in the periphery of HCs (n = 42), SLE (n = 28) and pemphigus patients (n = 94). Each symbol reflected one sample. Each bar indicated as mean ± S.E.M. **(c)** CD19^+^ and CD19^hi^ B cell population in human tonsils. Similar results were obtained in five independent experiments. ***P < 0.001.

CD19^hi^ B cells were reported previously in certain systemic autoimmune diseases, such as common variable immunodeficiency (CVID)^13^, SLE^14,15^, antineutrophil cytoplasm antibodies (ANCA)-associated vasculitis^14^ and scleroderma^16,17^. Herein, we not only validated the presence of CD19^hi^ B cell population in the periphery of SLE patients, but also observed them in the periphery of pemphigus patients, one of the organ-specific antibody-driven autoimmune diseases (Fig. 1a). CD19^hi^ B cells possessed larger size and increased granularity. The frequencies of CD19^hi^ B cells in SLE and pemphigus patients were dramatically higher than that in HCs (*p* < 0.001) (Fig. 1b), suggesting the aberrant distribution of CD19^hi^ B cells in both autoimmune diseases.

Not only in the periphery, CD19^hi^ B cells were also detectable in human secondary lymphoid organs as well. Human tonsils were obtained from either obstructive sleep apnea-hypopnea syndrome (OSAHS) or tonsillitis patients for CD19^hi^ B cell analysis. Results showed that nearly 50% of lymphocytes in human tonsils were CD19^+^ B cells where 10% of which were CD19^hi^ (Fig. 1c), indicating the prevalence of CD19^hi^ B cells both in the periphery and lymphoid organs.

**Figure 2:**
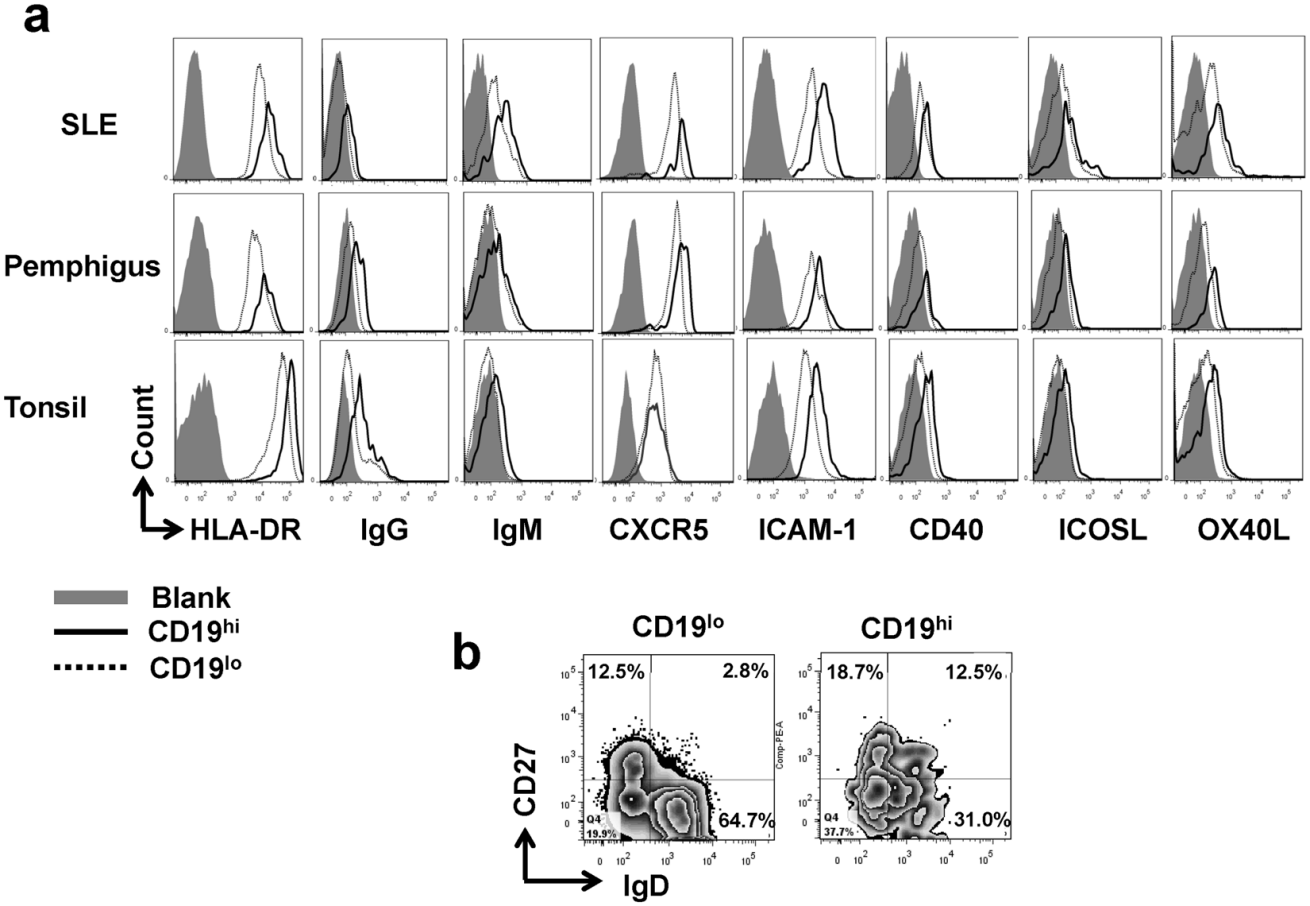
CD19^hi^ B cells display activated and memory-like phenotypes. (**a)** Comparison of HLA-DR, IgG, IgM, CXCR5, ICAM-1, CD40, ICOSL and OX40 L expression between CD19^hi^ and CD19^lo^ B cells from the periphery of SLE and pemphigus patients, as well as in human tonsil. (The gray area: blank control; solid line: CD19^hi^ B cells; dotted line: CD19^lo^ B cells). **(b)** CD27 and IgD expression in CD19^hi^ and CD19^lo^ B cells from human tonsil. Similar results were obtained from more than five independent experiments.

We further detected the expression of multiple molecules on cell surface to define the properties of CD19^hi^ B cells, including those responsible for the activation, costimulation and maturation (Fig. 2). Our results revealed that CD19^hi^ B cells in the periphery of SLE and pemphigus patients as well as in human tonsils exhibited an activated phenotype, demonstrated by the upregulation of HLA-DR, IgG and IgM expression. Molecules involved in costimulation, including ICAM-1, ICOSL, CD40 and OX40L, were also over-expressed in CD19^hi^ B cells when compared to CD19^lo^ counterparts. CXCR5 expression was higher in CD19^hi^ B cells from the periphery of SLE and pemphigus patients but not from tonsil (Fig. 2a). Consistent with the previous studies^14,15^, CD19^hi^ B cells contained more memory-like (CD27^+^) and memory/plasma-like (IgD^−^CD27^+^) cells but fewer IgD^+^CD27^−^ naïve B cells compared to CD19^lo^ B cells (Fig. 2b).

Therefore, CD19^hi^ B cells existing in the periphery of SLE and pemphigus patients as well as in tonsils belong to a distinct population with activated and memory-like phenotypes.

### CD19^hi^ B cells can be induced *in vitro* with the help of activated CD4^+^ T cells

**Figure 3:**
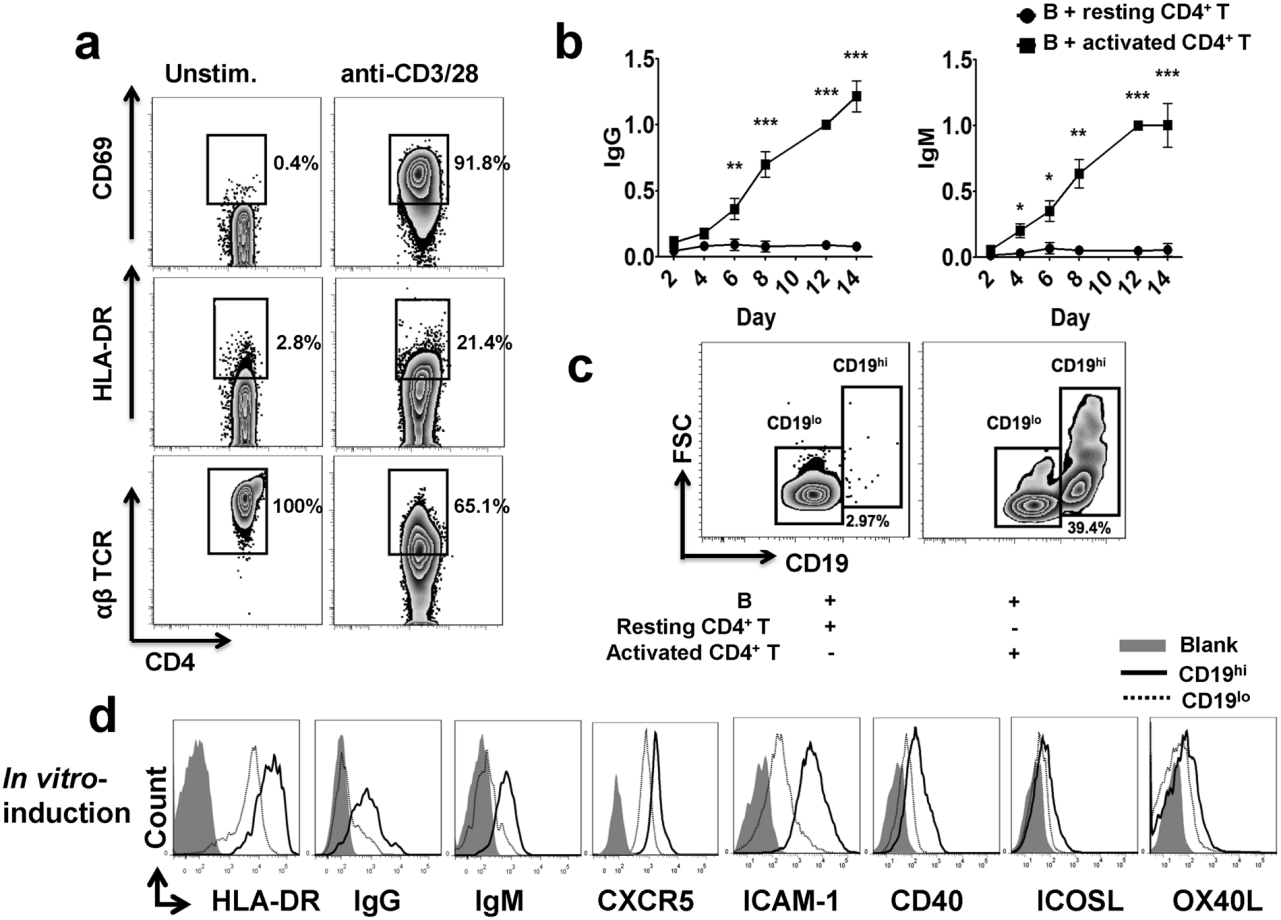
CD19^hi^ B cells can be induced *in vitro* with the help of activated CD4^+^ T cells. CD4^+^ T cells were purified from the periphery of HCs, and stimulated with or without human T-Activator CD3/CD28 Dynabeads for 24 hrs. **(a)** Representatives of CD69, HLA-DR and αβ TCR expression on resting and CD3/CD28 stimulated CD4^+^ T cells. **(b)** IgG (n = 12; left) and IgM (n = 7; right) production in the supernatants in cultures of B cells with autologous resting or activated CD4^+^ T cells on day 2, 4,6, 8, 12, and 14. The amount of IgG or IgM produced on day 12 was used for normalization in each individual sample. (bar: mean ± SEM), *P < 0.05; **P < 0.01;***P < 0.001. **(c)** Representatives of CD19^+^ B cell subpopulations cultured with resting or activated CD4^+^ T cells on day 12. Similar results were obtained in more than 12 independent experiments. **(d)** Detection of HLA-DR, IgG, IgM, CXCR5, ICAM-1, CD40, ICOSL and OX40 L expression on CD19^hi^ and CD19^lo^ B cells from *in vitro*-induction. The gray area: blank control; solid lines: CD19^hi^ cells; dotted lines: CD19^lo^ B cells. The data were representative of at least three independent experiments.

Although the presence of CD19^hi^ B cells was reported here as well as in the previous studies, how this population is generated is not clearly addressed. Considering the fact that activation of naïve B cells requires the help of CD4^+^ T cells, we established an *in vitro* CD4^+^ T and B cells co-culture system to mimic the bilateral T-B cell interaction. Purified human CD4^+^ T cells were stimulated *in vitro* with anti-CD3/CD28 Dynabeads for 24 hrs and co-cultured with autologous B cells later on. CD4^+^ T cells were fully activated with the dramatic increase of CD69 (early activation marker) and HLA-DR (late activation marker) expression as well as down-regulation of TCRαβ (Fig. 3a). When co-culturing activated CD4^+^ T cells with freshly isolated B cells, both IgG and IgM started to be detectable in the supernatants after 4 days, and dramatically increased along with the culture time (P < 0.001). However, little IgG and IgM were detected in the culture containing resting CD4^+^ T cells and B cells (Fig. 3b). These results indicated that activation of CD4^+^ T cells facilitated the differentiation of naïve B cells into immunoglobulin secreting cells.

We simultaneously analyzed the phenotypes of B cells after the co-culture. A CD19^hi^ B cell subpopulation with larger size and increased granularity was detectable after the co-culture with activated CD4^+^ T cells. However, very few CD19^hi^ B cells were found in the co-culture with resting CD4^+^ T cells (Fig. 3c). Similar to the results from SLE and pemphigus patients (Fig. 2), both activation markers (HLA-DR, IgG and IgM) and costimulatory molecules (including ICAM-1, CD40, ICOSL and OX40L) exhibited higher expression patterns on CD19^hi^ B cells when compared to CD19^lo^ counterparts (Fig. 3d), even more dramatically than those from SLE and pemphigus patients.

These results indicate that CD19^hi^ B cells can be induced *in vitro* with the help of activated CD4^+^ T cells, which probably recapitulates the *in vivo* generation of this population in SLE and pemphigus.

### CD19^hi^ B cells possess more capability to produce immunoglobulin

**Figure 4:**
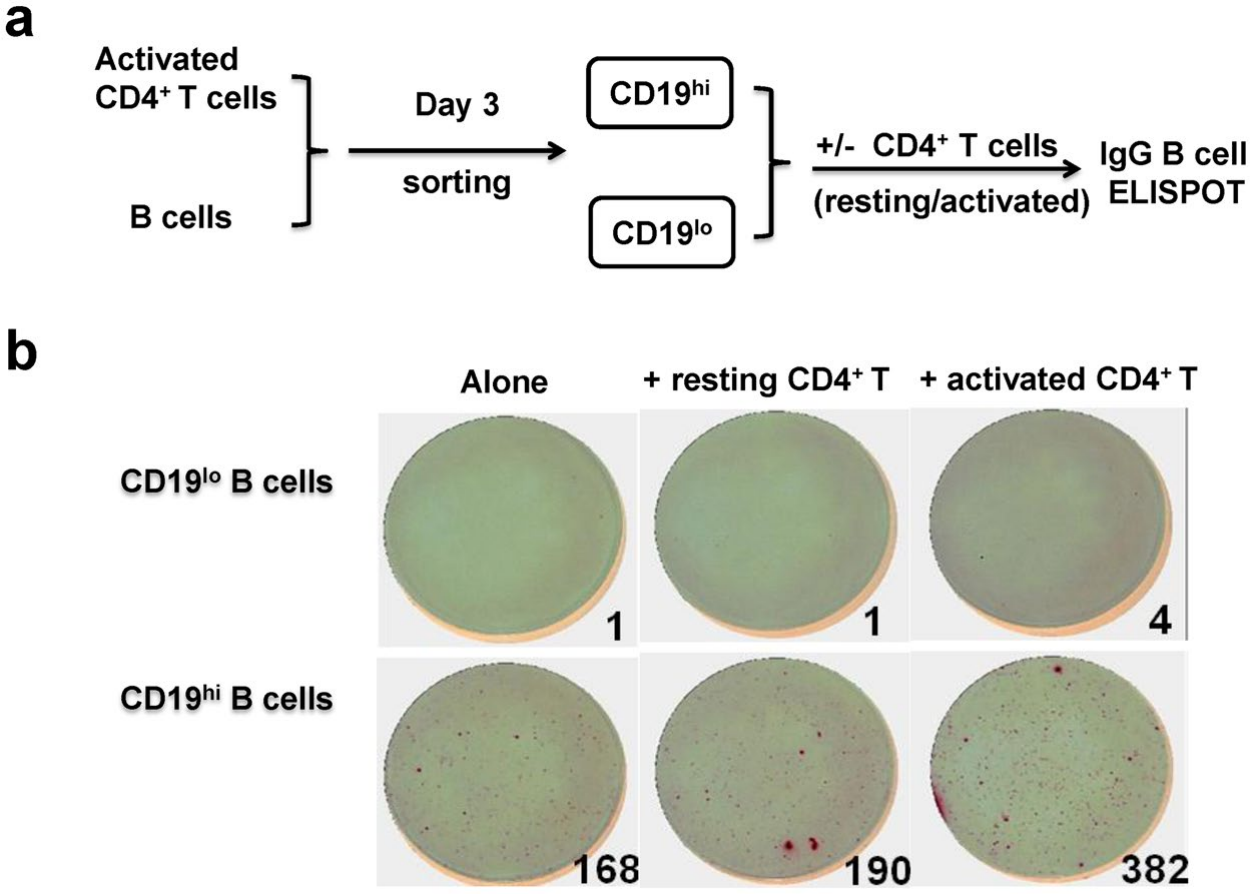
CD19^hi^ B cells possess more ability of immunoglobulin production. **(a)** Working scheme of detection on IgG production by CD19^hi^ and CD19^lo^ B cells. After 3 days’ co-culture with activated CD4^+^ T cells, CD19^+^ B cells differentiated into CD19^hi^ and CD19^lo^ B cells. Purified CD19^hi^ and CD19^lo^ B cells were then incubated for another 5 days with/without resting or activated CD4^+^ T cells, respectively. IgG ELSPOT assay was performed and SFU indicated the number of IgG-producing B cells in the culture. **(b)** Representative of IgG ELISPOT assay from CD19^hi^ and CD19^lo^ B cells. Upper lane: CD19^lo^ B cells alone, or co-cultured with resting or activated CD4^+^ T cells; Lower lane: CD19^hi^ B cells alone, or co-cultured with resting or activated CD4^+^ T cells. Similar results were obtained in two independent experiments.

Our above results indicated that activated CD4^+^ T cells triggered the generation of CD19^hi^ B *in vitro* with more IgG and IgM production. We continued to investigate the ability of CD19^hi^ and CD19^lo^ B cells to produce immunoglobulin by using an IgG ELISPOT assay. Purified CD19^+^ B cells were co-cultured with autologous activated CD4^+^ T cells *in vitro*. After 3 days, CD19^hi^ and CD19^lo^ B cells were sorted and incubated alone or co-cultured with resting or activated CD4^+^ T cells, respectively, for another 5 days (Fig. 4a). It was obvious that after the incubation more IgG-producing B cells were observed in the group of CD19^hi^ B cells with activated CD4^+^ T cells (Fig. 4b, low and right) than that with resting CD4^+^ T cells (Fig. 4b, low and middle). Interestingly, IgG-producing cells were comparable between CD19^hi^ B cells alone (Fig. 4b, low and left) and CD19^hi^ B cells co-culturing with resting CD4^+^ T cells (Fig. 4b, low and middle). On the contrary, very few IgG-producing cells were detected in the cultures of either CD19^lo^ B cells alone or those with two types of CD4^+^ T cells (Fig. 4b, upper panel).

The results indicate that CD19^hi^ B cells have more potential to produce immunoglobulin with the help of activated CD4^+^ T cells.

### CD19^hi^ B cells highly express genes involved in multiple signaling pathways for B-cell activation and differentiation

**Figure 5:**
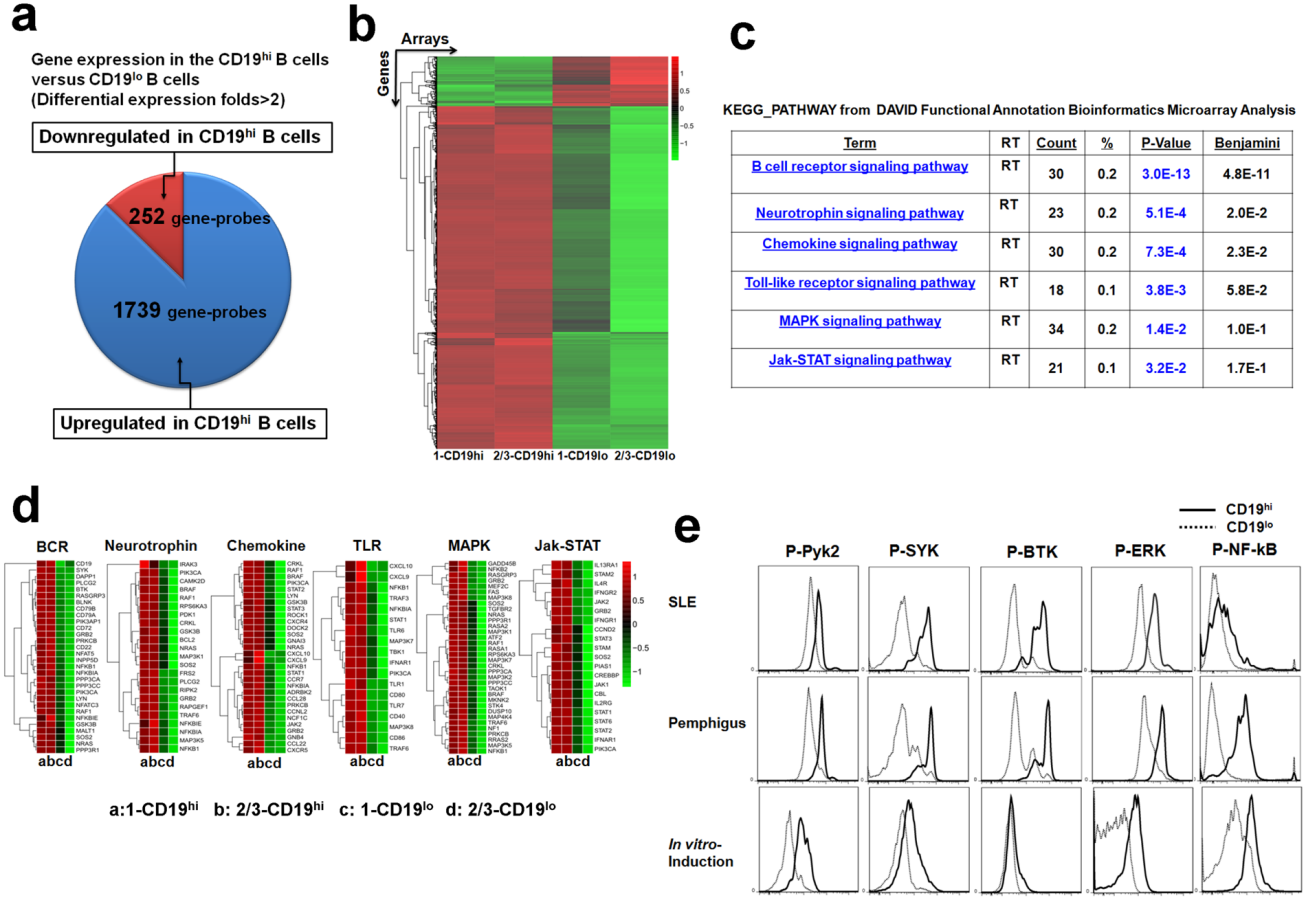
CD19^hi^ B cells highly express genes involved in multiple signaling pathways for B-cell development and activation. **(a)** CD19^+^ B cells isolated from the periphery of HCs were co-cultured with autologous stimulated CD4^+^ T cells for 3 days. CD19^hi^ and CD19^lo^ B cells were purified and subjected to genome-wide mRNA transcriptome microarray. Pie charts illustrated the number of differentially expressed genes between CD19^hi^ and CD19^lo^ B cells (signal intensity change ≥2-fold). **(b)** Heatmap of differentially expressed genes. Each line represented one gene probe. Each column represented one array. Different color represented the expression levels (from green to red: increased expression in CD19^hi^ B cells). **(c)** The layout of functional annotation chart from DAVID showing signaling pathways for upregulated genes by KEGG_PATHWAY. The most significantly enriched signaling pathways and their association values were listed. **(d)** Heatmaps indicating genes enriched in signaling pathways in CD19^hi^ and CD19^lo^ B cells determined by microarray. An increase or decrease of gene transcription by more than two-fold signal intensity was represented in red or green, respectively. Genes shown in black indicated no change in transcriptional abundance. **(e)** Representatives of phosphorylation levels of key signaling molecules including Syk, Erk, Btk, NF-κB and Pyk2 in *in vitro*-induced CD19^hi^ and CD19^lo^ B cells. (Solid lines: CD19^hi^ B cells; dot lines: CD19^lo^ B cells). The data were representative of at least three independent experiments.

Activation of CD4^+^ T cells facilitated naïve B cells to differentiate into CD19^hi^ B cells with more ability to produce IgG and IgM. What are the molecular mechanisms responsible for functional diversity between CD19^hi^ and CD19^lo^ B cells? Genome-wide microarray assay was conducted to define the transcriptome characters of these two cell subpopulations. There were 1,739 genes up-regulated and 252 genes down-regulated in CD19^hi^ B cells versus CD19^lo^ counterparts (Fig. 5a and b). Functional enrichment of differentially expressed genes by Kyoto Encyclopedia of Genes and Genomes (KEGG) pathway analysis showed that genes up-regulated in CD19^hi^ B cells were enriched in multiple signaling pathways involved in B cell activation and differentiation, including B cell receptor (BCR), chemokine, Toll-like receptor (TLR), MAPK and Jak-STAT signaling (Fig. 5c). Most of representative genes involved in these signaling pathways were up-regulated in CD19^hi^ B cells based on the microarray analysis, which played diverse roles in B-cell ontogeny, activation, differentiation, survival and antibody production (Fig. 5d). Phosphorylation of signaling molecules involved in BCR signaling were further analyzed in CD19^hi^ and CD19^lo^ B cells from patients or induced *in vitro*. It was indicated that the phosphorylation levels of signaling molecules, including Pyk2, Syk, Erk, Btk and NF-κB in CD19^hi^ B cells were dramatically higher than those in CD19^lo^ B cells (Fig. 5e).

Therefore the results from genome-wide transcriptome analysis and phosphorylation profiling assay further confirm the activation property of CD19^hi^ B cells.

### More CD19^hi^ B cells are induced *in vitro* by fresh CD4^+^ T cells from SLE and pemphigus patients with elevated IgG production

**Figure 6:**
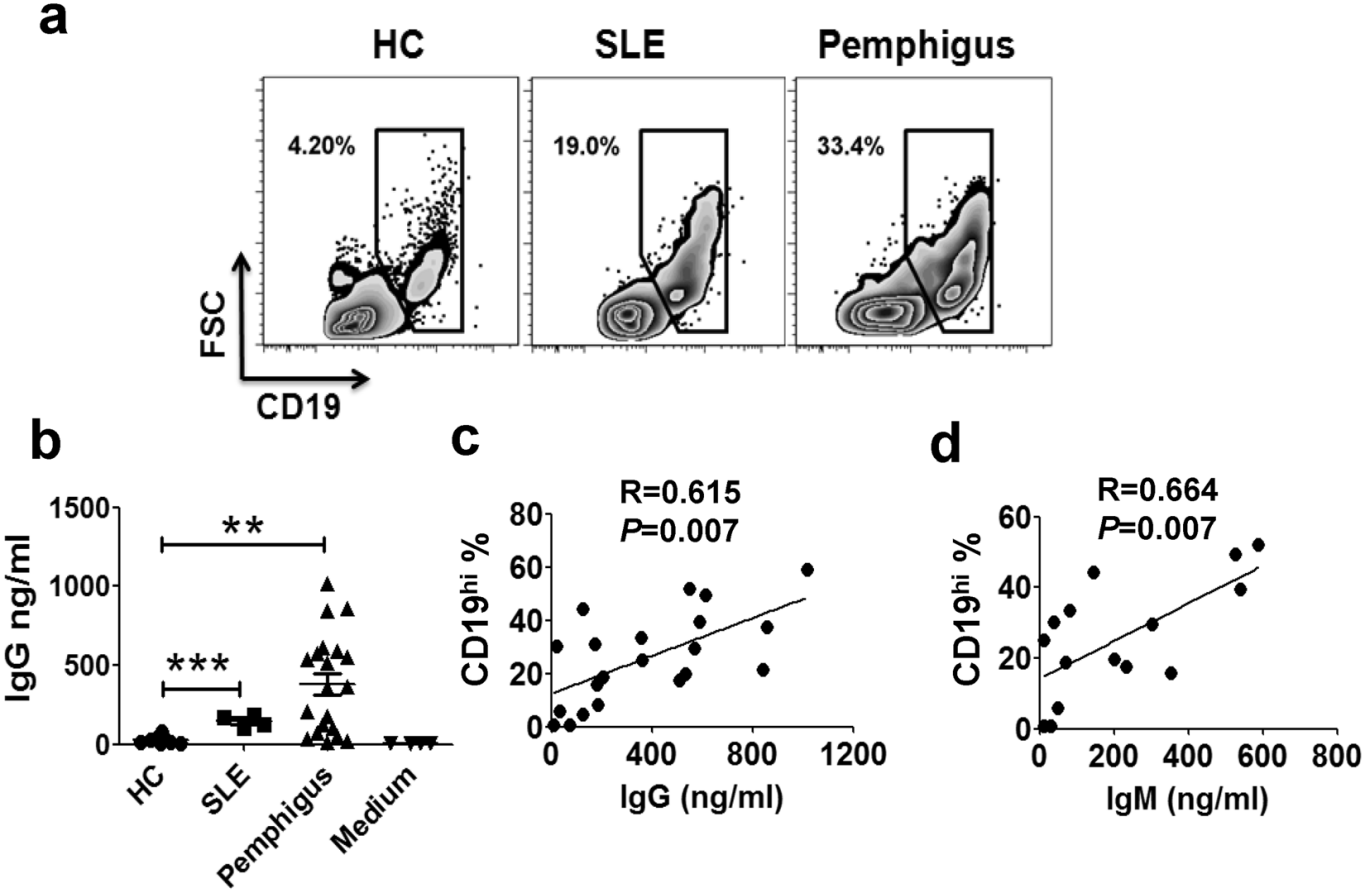
More CD19^hi^ B cells are induced *in vitro* by fresh CD4^+^ T cells from SLE and pemphigus patients with elevated IgG production. (**a**) Representatives of CD19^hi^ B cells after co-culturing B cells with autologous fresh CD4^+^ T cells from the periphery of HC, SLE and pemphigus, respectively. (**b**) IgG production in the supernatants of B cells co-culturing with autologous fresh CD4^+^ T cells from HC (n = 10), SLE (n = 4) and pemphigus (n = 20). Each bar indicated mean ± SEM. **P < 0.01; ***P < 0.001. (**c**–**d**) Associations of the frequencies of *in vitro*-induced CD19^hi^ B cells with supernatant IgG (**c**) (n = 21) and IgM (**d**) (n = 15) levels in pemphigus patients.

Our above results demonstrated that activation of CD4^+^ T cells from HCs promoted the generation of CD19^hi^ B cells *in vitro* (Fig. 3). Since CD4^+^ T cells in patients possessed an activated phenotype with dramatic increase of CD69 expression, which was strongly and positively correlated with CD19^hi^ B cell frequency (Supplementary Fig. 1), we further investigated the possibility whether higher percentages of CD19^hi^ B cells in the periphery of SLE and pemphigus patients (Fig. 1) were derived from the help of activated CD4^+^ T cells under the pathology. Freshly isolated B cells from SLE or pemphigus patients were co-cultured with autologous CD4^+^ T cells. It was found that more CD19^hi^ B cells were induced in the cultures from SLE and pemphigus patients when compared to HCs after 12 days (Fig. 6a). Consistent with the increase in the percentages of CD19^hi^ B cells, more IgG was produced in the supernatants of CD4^+^ T-B co-culture from the patients (Fig. 6b). In addition, the frequency of CD19^hi^ B cells after the co-culture was closely associated with the levels of both IgG and IgM in the supernatants (Fig. 6c and d). Higher frequencies of CD19^hi^ B cells existing in SLE and pemphigus patients thus might be due to strong capacity of pathogenic CD4^+^ T cells to trigger B cell differentiation.

### Interactions between costimulatory molecules contribute to CD19^hi^ B-cell generation and antibodies production

**Figure 7:**
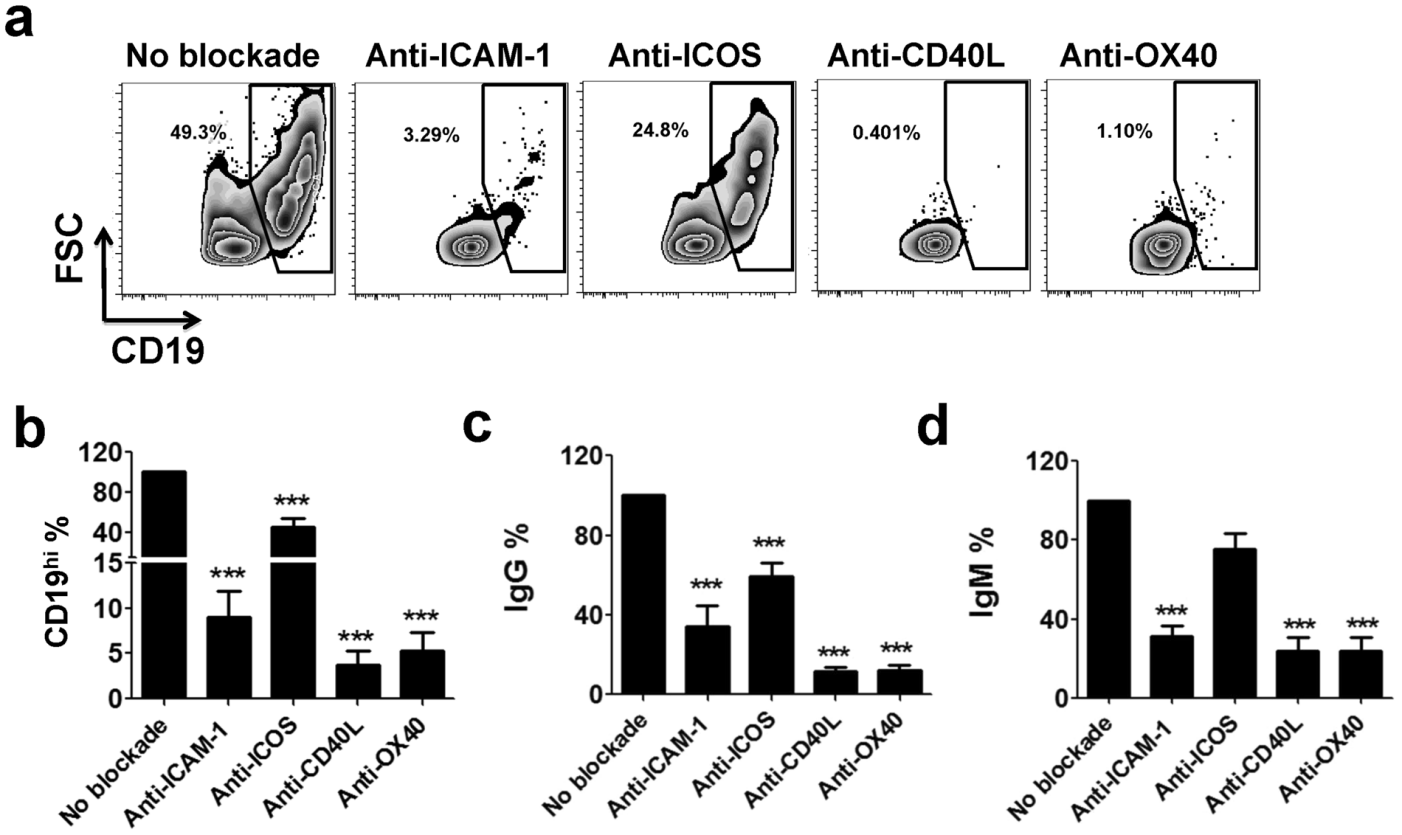
Interactions between costimulatory molecules contribute to CD19^hi^ B-cell generation and antibodies production. Freshly isolated CD4^+^ T were co-cultured with autologous B cells from pemphigus patients (n = 10). Functional blockade antibodies, including anti-ICAM-1, anti-CD40L, anti-OX40 and anti-ICOS were added during the co-culture. On day 12, the percentage of CD19^hi^ B cells was evaluated by flow cytometry **(a,b)**, and the IgG and IgM levels in supernatants were determined by ELISA according to the manufacturer’s instructions **(c,d)**.

Costimulatory molecules (including ICAM-1, CD40, ICOSL and OX40L) have been demonstrated to play an important role in facilitate T/B cell interaction as well as B cell activation and differentiation^18^. Since multiple costimulatory molecules were observed to be highly expressed in CD19^hi^ B cells (Figs 2 and 3), we further investigated whether the interactions between costimulatory molecules such as ICAM-1-LFA1, ICOS-ICOSL, CD40-CD40L and OX40-OX40L were involved in CD19^hi^ B-cell generation. Functional blockade antibodies were added when co-culturing fresh CD4^+^ T with autologous B cells from pemphigus patients. Our data showed that the addition of antibodies targeting costimulatory molecules ICAM-1, CD40L, ICOS or OX40 dramatically attenuated CD19^hi^ B-cell generation as well as IgG and IgM production (Fig. 7). These data indicate that interactions between costimulatory molecules mostly contribute to the generation of CD19^hi^ B-cell and IgG/IgM production.

### Frequencies of peripheral CD19^hi^ B cells from SLE and pemphigus patients are correlated with serum total IgG and IgM, but not with autoantibodies and disease severity

**Figure 8:**
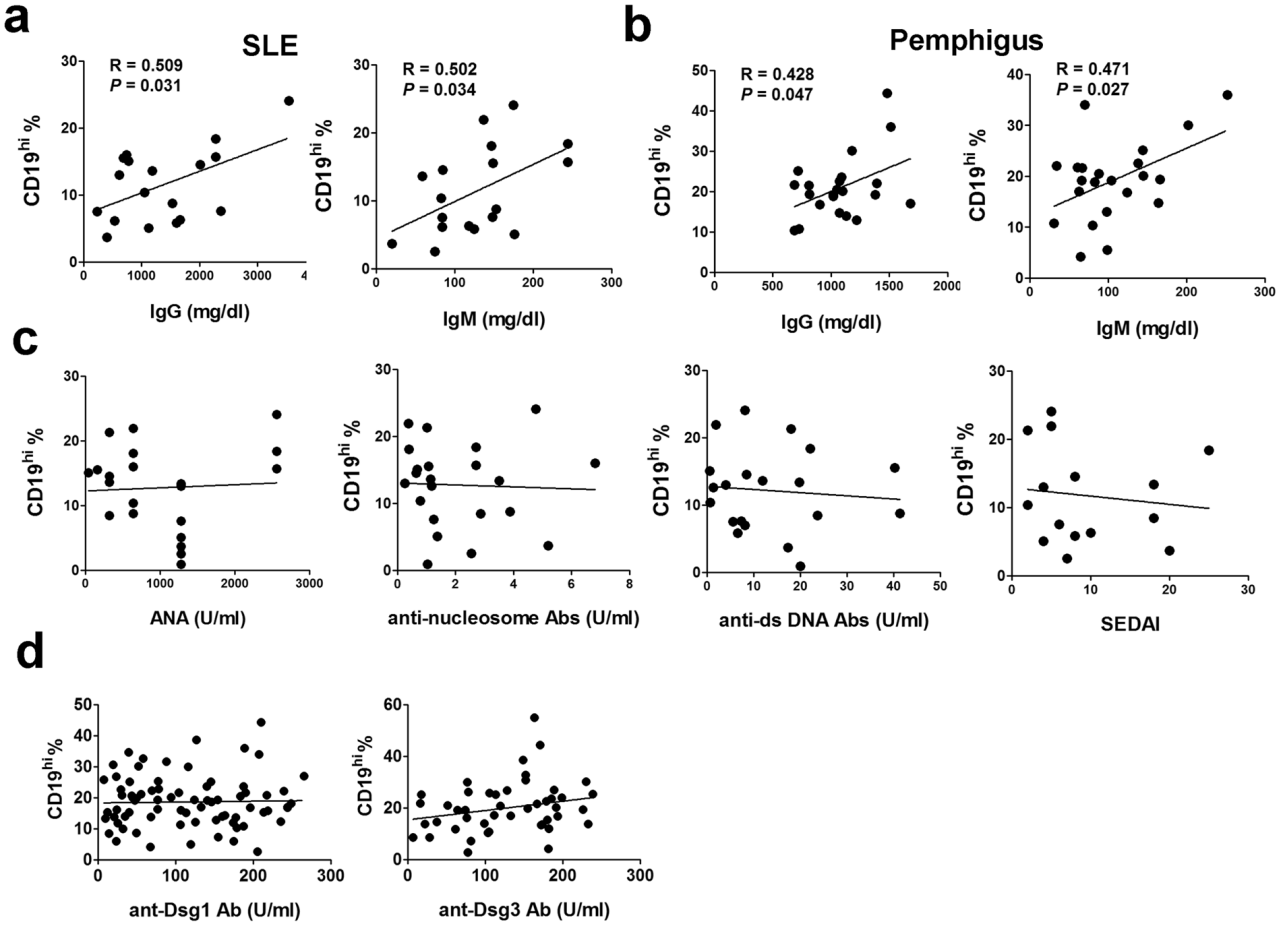
Frequencies of peripheral CD19^hi^ B cells from SLE and pemphigus patients are correlated with serum total IgG and IgM, but not with autoantigen-specific antibodies and severity. (**a**,**b**) Correlations between the frequency of circulating CD19^hi^ B cells with serum total IgG and IgM levels in SLE (**a**) and pemphigus patients (**b**). (**c**) Correlations between circulating CD19^hi^ B cells with ANA, anti-nucleosome and anti-ds DNA antibodies, and SEDAI in SLE. (**d**) Correlations between the frequency of circulating CD19^hi^ B cells with anti-Dsg1 and Dsg3 autoantibodies in pemphigus.

To further elucidate the clinical significance of CD19^hi^ B cells in SLE and pemphigus, we performed the correlation analysis between the percentage of CD19^hi^ B cells and clinical parameters related to the diseases diagnosis and severity, including serum total IgG/IgM, and anti-nuclear antibody (ANA), anti-nucleosome antibody, anti-double strand DNA (anti-dsDNA) antibody and SEDAI (Systemic Lupus Erythematosus Disease Activity Index) for SLE, as well as anti-Dsg1/Dsg3 antibodies for pemphigus. Our results revealed that the frequencies of CD19^hi^ B cells were strongly correlated with serum total IgG and IgM levels in both SLE (Fig. 8a) and pemphigus (Fig. 8b). However, no correlation was observed between the frequency of CD19^hi^ B cells and the levels of ANA, anti-nucleosome or anti-ds DNA antibodies in SLE. Neither was observed with SLEDAI index (Fig. 8c). In pemphigus, serum anti-Dsg1 and Dsg3 autoantibodies levels were not related to peripheral CD19^hi^ B cells either (Fig. 8d). Our clinical correlation analysis thus largely validates the important contribution of CD19^hi^ B cells to total IgG and IgM production observed in both *in-vivo* and *in-vitro* system.

## Discussion

Abnormal B-cell activation and differentiation in antibody-driven autoimmune diseases is one of the hallmarks with the continuous production of autoantibodies. In the present study, we have defined a CD19^hi^ B cell subset as a key contributor to total IgG and IgM production in SLE and pemphigus with the unique phenotypes and functionality.

The presence of CD19^hi^ B cells was previously reported in SLE^14,15^. Consistent with these studies^14,15^, we validated the presence of CD19^hi^ B cells in the periphery of SLE exhibiting activation and memory-like phenotypes (Figs 1 and 2). This B cell subpopulation also possesses strong ability to produce IgG and IgM (Figs 6 and 8). In addition, we also observed the high proportion of CD19^hi^ cells in the periphery of pemphigus patients, which is an organ-specific autoantibody-driven autoimmune disease (Fig. 1). Through performing transcriptome analysis of CD19^hi^ B cells, we have identified a number of important genes involved in the function of this cell subset (Fig. 4). Upregulation of costimulatory molecules, such as ICAM-1, ICOS, CD40L and OX40 on CD19^hi^ B cells (Figs 2 and 3), contributed to the CD19^hi^ B-cell generation (Fig. 7). In addition, more CD19^hi^ B cells were generated upon anti-IgM stimulation as well (Supplementary Fig. 2). What is more, frequencies of peripheral CD19^hi^ B cells from SLE and pemphigus patients were correlated with serum total IgG and IgM, but not with autoantigen-specific antibodies and disease severity (Fig. 8). Therefore, our study strongly implies that CD19^hi^ B cells contain B cell precursors for terminal differentiation and contribute to total IgG/IgM production in human autoimmune diseases. Whether antigen-specific BCR stimulation or other molecules are involved in promoting terminal differentiation of CD19^hi^ B cells needs to be further investigated.

High expression of CD19 is the most apparent character of this B cell subset. CD19 is a BCR co-receptor that positively regulates BCR signaling through forming a multimeric protein complex with CD21 and CD81^19^. Cross-linking of CD19 and BCR results in rapid phosphorylation of cytoplasmic tail of Lyn, which triggers downstream signaling pathways including Akt-PI3K and MAPKs signaling^20–23^, and subsequent B cell activation. Therefore, CD19 serves as a co-stimulatory molecule to reduce the threshold for B cell activation^24^. Alteration in CD19 expression is reported to be able to shift the balance between tolerance and immunity, leading to autoimmunity^25^. Transgenic mice with 15–29% increase in CD19 expression on cell surface displayed the loss of tolerance and the spontaneous generation of antinuclear autoantibodies, rheumatoid factor, and autoantibodies against ssDNA, dsDNA and histone^16^. Peripheral B cells from systemic sclerosis patients had about 20% higher of CD19 density compared to that from normal individuals^16^. Previous studies on the introduction of CD19^hi^ B cell subpopulation highlight the roles of CD19 in regulating B cells homeostasis during the onset and relapse of autoimmune diseases^13–16,25–27^. In our study, high expression of CD19 on this population is companied with the hyper-expression of activation markers, including increased granularity and higher levels of HLA-DR, IgG and IgM (Figs 1–3), which is consistent with activated phenotypes reported previously^14^. What is more, multiple costimulatory ligands, including ICAM-1, ICOSL, CD40 and OX40L (Figs 2 and 3) were also up-regulated. Since these co-stimulatory signals facilitate T/B cell interaction as well as B cell activation and differentiation^18^, it is not out of expectation that CD19^hi^ B cells possess more ability to produce more IgG in our study (Fig. 4). Combining the results from phenotypic and functional studies, we suggest that high expression of CD19 together with high levels of costimulatory molecules on this B cell subpopulation make CD19^hi^ B cells more susceptibility to stimuli together with stronger ability to produce IgG and IgM.

Results from global gene expression profiling provide transcriptional evidence on the activation of CD19^hi^ B cells simultaneously (Fig. 5). Genes involved in BCR, neurotrophin, chemokine, TLR, MAPK and Jak-STAT signaling pathways (Fig. 5c and d) were up-regulated in CD19^hi^ B cells, which play indispensable roles in B-cell activation, differentiation, and antibody production^28,29^. For instance, the expression of genes involved in BCR signaling, such as *CD79A, CD79B, LYN, SYK, BTK, PI3K, PLCγ2, CD22* and *CD72*, was dramatically enhanced in CD19^hi^ B cells when compared to CD19^lo^ counterparts (Fig. 5d). Some of them were validated through determining their phosphorylation levels by FACS analysis, including Syk and ERK1/2 (Fig. 5e), which corroborate previous findings^15^. In addition, up-regulation of genes related to neurotrophin in CD19^hi^ B cells (Fig. 5c,d) might be associated with the previous findings showing that SLE patients with CD19^hi^ B cells had a greater frequency of neurologic complications^14,15^. Several chemkines (CC), such as *CXCL9*, *CXCL10*, *CCL22*, *CCL28*, and CC ligands such as *CXCR4* and *CXCR5*, were up-regulated in CD19^hi^ B cells in transcriptome assay as well (Fig. 5 and Supplementary Fig. 3). Among them, up-regulation of CXCR5 on CD19^hi^ B cells was validated by flow cytometric assay either from the periphery of patients or *in-vitro* induction (Figs 2 and 3). Being consistent with the transcriptomic data, CD19^hi^ B cells exhibited a high migration activity in response to CXCR4 and CXCR5 stimulation *in vitro*, but not to CXCR3 stimulation (Supplementary Fig. 3).

It is observed that there are more CD19^hi^ B cells detectable in the periphery of SLE and pemphigus patients (Fig. 1). We are very interested in defining the origin of this B cell subset. Through establishing a co-culture system *in vitro* with autologous CD4^+^ T and B cells from HCs, we have successfully induced CD19^hi^ B cell population *in vitro*. They possessed the similar phenotypes with those from SLE and pemphigus patients (Figs 2 and 3). More importantly, this occurs only when activated CD4^+^ T cells are involved. Peripheral CD4^+^ T cells from SLE and pemphigus patients, which are in an activation status (Supplementary Fig. 1), reinforced the generation of CD19^hi^ B cells after *in vitro* co-culture (Fig. 6a). It is the first to report that the CD19^hi^ B cells are induced with the help of activated CD4^+^ T cells either *in vitro* or *in vivo*.

Clinical significance of this unique CD19^hi^ B cell subpopulation in antibody-driven autoimmune diseases was investigated in this study as well. SLE and pemphigus, although rarely clinically associated, share similar mechanisms for the loss of B-cell tolerance and the presence of autoantibodies in high titers. With the presence of high percentage of CD19^hi^ B cells in the periphery of SLE and pemphigus, we once speculated that they might be strongly correlated with autoantibody levels or disease severity. However, it is not the case. No correlations were observed between the frequency of peripheral CD19^hi^ B cells and the levels of ANA, anti-nucleosome and anti-ds DNA antibodies as well as SEDAI in SLE patients (Fig. 8c). Neither was observed in pemphigus between the proportion of CD19^hi^ B cells and anti-Dsg1 or anti-Dsg3 autoantibodies (Figs 1 and 8d). The percentage of CD19^hi^ B cells was only positively correlated with the levels of total IgG and IgM in the supernatants or in the serum (Figs 6 and 8), which suggests that CD19^hi^ B cells are more likely to contain the precursors of anti-self PCs which will be further enriched in autoreactivity.

In fact, the increased IgG and IgM have been observed in certain B cell lymphoma, such as diffuse large B-cell lymphomas (DLBCL)^30^. DLBCL can be subtyped into germinal centre B-cells (GCB)-like DLBCL and activated B-cells (ABC)-like DLBCL based on gene expression profiles. GCB-DLBCL is characterized by the expression of genes similar to normal GC B cells while ABC-like DLBCL possess the gene panels similar to *in vitro* activated peripheral blood B cells^31–33^. CD19^hi^ B cells identified in our study are more apt to the property of ABC-like DLBXL with similar gene expression profiles (data not shown). There are studies reporting the higher risk of malignancies in SLE patients, such as non-Hodgkin lymphoma and Hodgkin lymphoma^34^. Whether the presence of CD19^hi^ B cells might be the risky factor or the predicative factor of malignancies need to be further investigated. In addition, it has been reported that a subgroup of SLE patients with CD19^hi^ B cells had severe clinical outcomes and a poor response to rituximab treatment^15^. A recent study on the relationship between serum Igs and the risk of infection indicated that low levels of IgG and/or IgM were associated with a heightened risk of infections^35^. Herein the percentage of CD19^hi^ B cells might be also useful to indicate the risk of infection.

Taken together, it is evident that there exist CD19^hi^ B cells in the periphery of SLE and pemphigus patients exhibiting activation and memory-like properties. They can be induced with the help of activated CD4^+^ T cells *in vitro*. With strong correlation between peripheral CD19^hi^ B cells and total IgG/IgM levels in SLE and pemphigus patients, CD19^hi^ B cells might represent a distinct B cell subset contributing to IgG/IgM production in human autoimmune diseases. Their clinical involvement in the progression and development of antibody-mediated autoimmune diseases needs to be further investigated.

## Materials and Methods

### Human subjects

A total of 34 SLE patients and 94 pemphigus patients were enrolled in this study from Ruijin Hospital affiliated to Shanghai Jiao Tong University School of Medicine. All SLE patients fulfilled the American Rheumatism Association criteria for the diagnosis of SLE. The diagnosis of pemphigus was confirmed by clinical manifestations, histology and at least one positive serological test (direct immunofluorescence, indirect immunofluorescence or Dsg 1/Dsg 3 ELISA) accordingly^36^. The study was approved by the Ethic Committee of Ruijin Hospital affiliated to Shanghai Jiao Tong University School of Medicine and all experiments were performed according to the principles of the Declaration of Helsinki. Informed consent forms were assigned individually. Healthy controls (n = 56) were from volunteers.

### CD4^+^ T cell activation and T-B cell co-culture *in vitro*

Whole blood was collected in heparin lithium-treated tubes. Peripheral blood mononuclear cells (PBMCs) were isolated by density gradient centrifugation using Lymphoprep^TM^ (Axis- shield, Norway)^37^. CD4^+^ T cells and B cells from HCs or patients were isolated by using human CD4^+^ T Cell Isolation Kit II and B Cell Isolation Kit II (Miltenyi Biotec, Germany), respectively, according to the manufacturer’s instructions. Purity of isolated CD4^+^ T cells and B cells was determined by flow cytometry. Cells with purity over 95% were used for further experiments.

For activation, CD4^+^ T cells from HCs were incubated with human T-Activator CD3/CD28 Dynabeads (Life Technologies, USA) (at a cell:bead ratio of 1:1) for 24 hrs in 96-well U-bottom plates (Costar, USA). Cells were harvested for T-B cell co-culture *in vitro* and flow cytometry analysis after the deletion of Dynabeads.

Resting or activated CD4^+^ T cells from HCs were co-cultured with autologous B cells (10^4^: 10^4^) in RPMI 1640 medium supplemented with 10% fetal bovine serum (FBS) (Life Technologies, USA) and penicillin-streptomycin (Life Technologies, USA). Supernatants were harvested on day 2, 4, 6, 8, 12 and 14, respectively, for IgG and IgM measurement. For SLE and pemphigus samples, B cells were co-cultured with freshly isolated autologous CD4^+^ T cells (2 × 10^4^: 5 × 10^4^). Supernatants were harvested on day 12 for IgG and IgM measurement. The remaining cells were subjected to phenotypic and functional analysis.

In blocking experiments, fresh isolated CD4^+^ T were co-cultured with autologous B cells (5 × 10^4^: 2 × 10^4^) from pemphigus patients, and different blocking antibodies including purified mouse anti-human CD54 (ICAM-(1), purified mouse anti-human CD154 (CD40L), purified mouse anti-human CD134 (OX40) (BD Biosciences, USA) and anti-human CD278 (ICOS) functional grade purified (eBioscience, USA) were added with a final concentration of 5 μg/ml.

### Flow cytometry

For phenotypic analysis, cells were resuspended in 100 μl staining buffer (PBS containing 3% FBS) and incubated with fluorochrome-conjugated monoclonal antibodies (mAbs) at room temperature (RT) for 30 min, including FITC-anti CD19, FITC-anti CD4, Pacific blue-anti CD4, PE-Cy5^TM^ -anti ICAM-1, APC-anti CD40L, PE-anti ICOSL, FITC-anti OX40 L (all above mAbs were from BD Biosciences, USA), PE-anti CD69 and PE-anti ICOS (eBioscience, USA). After washing once with staining buffer, cells were resuspended in 200 μl PBS with 2% paraformaldehyde (PFA) before acquisition.

Phosphorylation of intracellular Syk, ERK, Btk, Itk and Pyk2 in B cells was determined by using Cytofix/Cytoperm^TM^ Plus Fixation/Permeabilization Kit (BD, USA). Briefly, cells were labeled with FITC- or PeCy5-anti CD19 mAbs first, and fixed and permeabilized by using Fix/Perm solution in the dark for 30 min at RT. After washing once with 1 × Perm/Washing buffer, cells were incubated with PE-anti Pyk2 (pY402), PE-anti Syk (pY348), FITC-anti Btk (pY551), FITC-anti Itk (pY511) and APC-anti ERK1/2 (pT202/pY204) (BD Biosciences, USA) for 1 hr at RT followed by washing once with 1 × Perm/Washing buffer. Cells were resuspended in 200 μl PBS with 2% PFA.

Phosphorylation of NF-κB in B cells was detected according to the manufacturer’s instruction of Foxp3/Transcription Factor Staining Buffer Set and One-step protocol for intracellular (nuclear) protein (eBioscience, USA). After staining with antibodies against surface markers, cells were fixed and permeabilized by Fix/Perm buffer for 1 hr at RT and washed once with 1 × Perm buffer. Cells were incubated with PE-Cy7-anti NF-κB p65 (pS529) (BD Biosciences, USA) for 1 hr at RT followed by washing with 1 × Perm buffer and resuspended in 200 μl PBS with 2% PFA.

Cells were acquired on BD FACS Canto II (BD Biosciences, USA) and data were analyzed by using Flowjo 7.6.1 software (FlowJo, USA).

### Cell sorting

After the co-culture of autologous CD4^+^ T and B cell for 3 days, cell mixture was labeled with FITC-anti CD19, PE-anti CD4 and PE-Cy5^TM^-anti ICAM-1 (BD Biosciences) for 30 min at RT. CD19^hi^ and CD19^lo^ B cells were sorted out on a FACS ARIA (BD Biosciences). Purity was verified by flow cytometry with at least 95% purity.

### IgG ELISPOT assay

Purified CD19^hi^ and CD19^lo^ B cells were incubated with or without autologous CD4^+^ T cells for 5 days at 37 °C. IgG B cell enzyme-linked immunospot (ELSPOT) assay (U-CyTech, Netherlands) was performed according to the manufacturer’s instruction. One spot forming unit (SFU) represented one antibody secreting cell (ASC).

### ELISA

Supernatants collected from T-B co-culture at different time points were subjected to enzyme-linked immunosorbent assay (ELISA) for IgG and IgM production according to the manufacturer’s instructions (Sen Xiong, Shanghai, China).

### Microarray assay

Total RNA from sorted CD19^hi^ and CD19^lo^ B cells of three HCs was extracted by using Trizol reagent (Invitrogen, USA) and purified using RNeasy Mini Kit (Qiagen, Germany). Microarray assay was performed by using Affymetrix GeneChip® Human Transcriptome Array 2.0 (HTA 2.0) (Affymetrix, Canada) following the Affymetrix one cycle target labeling protocol by Shanghai SBS Company (Shanghai, China). Genes with signal intensity changes ≥2 folds between CD19^hi^ and CD19^lo^ B cells were considered as differentially expressed. Heatmaps of differentially expressed genes were generated with MultiExperiment Viewer software (MeV). Functional annotation of genes of interests was carried out with DAVID Bioinformatics Resources (http://david.ncifcrf.gov/home.jsp). The complete microarray data of CD19^hi^ and CD19^lo^ B cells can be found at the NCBI Gene Expression Omnibus with accession number GSE96600.

### Statistical analysis

Data were presented as means ± standard error of means (S.E.M)^38,39^. Statistical analyses were performed by using Graphpad Prism 5.0 software (Graphpad software Inc. USA). Statistic difference was determined by unpaired *Student t* test for the data with gaussian distribution, and by Mann-Whitney test for those with non-gaussian distribution. Unless stated, *p* < 0.05 was considered statistically significant.

## Electronic supplementary material

Supplemental Materials
