# Supplementary material for: Peripheral CD19hi B cells exhibit activated phenotype and functionality in promoting IgG and IgM production in human autoimmune diseases
Source: Sci Rep. 2017 Oct 24;7:13921. doi: 10.1038/s41598-017-14089-2 (PMC5655037; doi:10.1038/s41598-017-14089-2)
Supplement: Supplementary file 1 — Supplemental Materials [file 41598_2017_14089_MOESM1_ESM.pdf]

Title page

Title: Peripheral CD19^{hi} B cells exhibit activated phenotype and functionality in promoting IgG and IgM production in human autoimmune diseases

Authors:

Zhicui Liu[1,2,#], Weihong Zeng[2,3,#], Xiangyang Huang[4,#], Shujun Wang[2], Jie Zheng[1], Meng Pan[1*] and Ying Wang[2*]

Institution:

[1] Department of Dermatology, Ruijin Hospital, Shanghai Jiao Tong University School of Medicine, 200025, Shanghai, P. R. China.

[2] Shanghai Institute of Immunology, Shanghai Jiao Tong University School of Medicine, 200025, Shanghai, P. R. China.

[3] Institute of Embryo-Fetal Original Adult Disease Affiliated to Shanghai Jiao Tong University School of Medicine, the International Peace Maternity & Child Health Hospital, Shanghai Jiao Tong University School of Medicine, 200030, Shanghai, P. R. China.

[4] Department of Rheumatology and Immunology, West China Medical School of Sichuan University, 610041, Chengdu, Sichuan, P. R. China.

#: These authors contributed equally to this work.

* **Corresponding authors:**

Ying Wang, Shanghai Institute of Immunology, Shanghai Jiao Tong University School of Medicine, 200025, Shanghai, P. R. China. E-mail: ywang@sibs.ac.cn;

Meng Pan, Department of Dermatology, Ruijin Hospital, Shanghai Jiao Tong University School of Medicine, 200025, Shanghai, P. R. China. E-mail: panmeng@medmail.com.cn.

Supplemental Materials and Methods

T-B cell co-culture and anti-IgM stimulation

Peripheral blood mononuclear cells (PBMCs) were isolated by density gradient centrifugation using Lymphoprep™ (Axis- shield, Norway) from pemphigus patients. $CD4^+$ T cells and B cells were isolated by using human $CD4^+$ T Cell Isolation Kit II (Miltenyi Biotec, Germany) and EasySep™ Human CD19 Positive Selection Kit II (STEMCELL Technologies, Canada), respectively, according to the manufacturer's instructions. Purity of isolated $CD4^+$ T cells and B cells was determined by flow cytometry. Cells with purity over 95% were used for further experiments.

Fresh isolated $CD4^+$ T were co-cultured with autologous B cells (3×10^5 : 1×10^5), and Goat $F(ab')^2$ anti-Human IgM, Fc5μ fragment specific, Unconjugated (Jackson, USA) was added with a final concentration of 20 μg/ml. After a 8-day incubation, the cells were subjected to flow cytometry analysis.

Transwell migration assay

Peripheral blood $CD4^+$ T cells and autologous B cells were isolated from pemphigus patients as described above. Isolated $CD4^+$ T cells and B cells (2×10^5 : 1×10^5) were resuspended in 300 μL RPMI-1640 medium containing 10% FBS, and were placed into the upper chambers of the transwell inserts (8-μm pore size; Corning, USA). 500 μL RPMI-1640 mediums (with 10% FBS) containing recombinant human MIG (CXCL9), SDF-1α (CXCL12) or BCA-1 (CXCL13; Peprotech, USA) at a final concentration of 250 ng/mL were loaded on the lower chambers of 24-well transwell

plates. After a 24-hour incubation, the cells in both upper and lower chambers were subjected to flow cytometry analysis.

Supplemental Figure

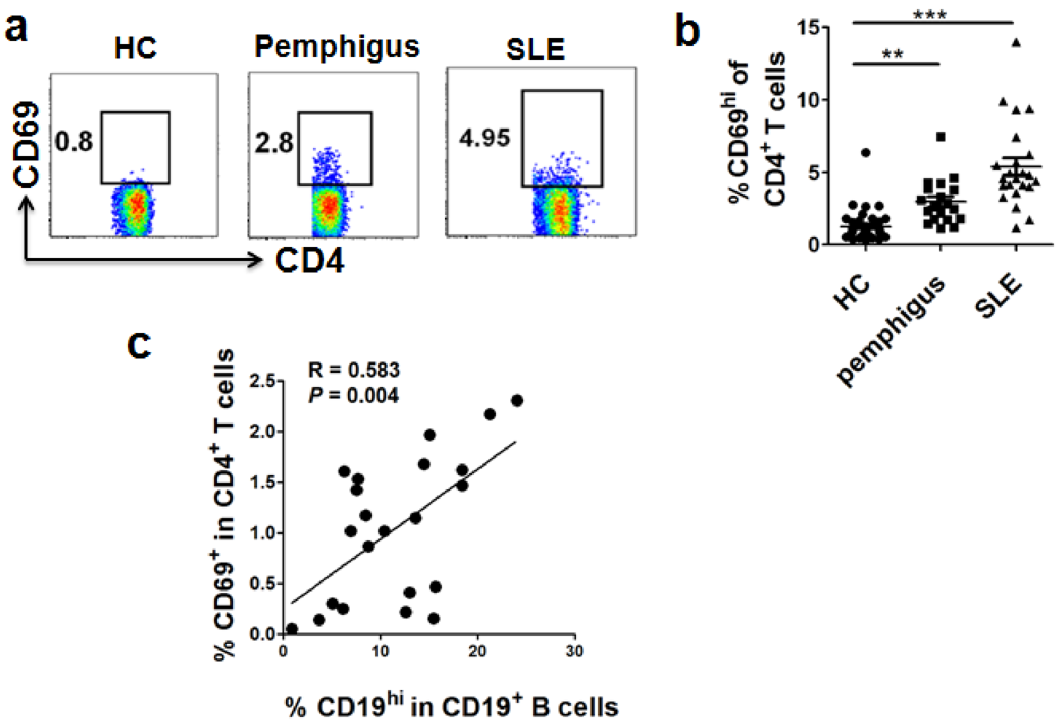

Figure S1. Peripheral CD4$^+$ T cells showed a higher expression of CD69 in SLE and pemphigus patients than in healthy controls. (a-b) Representative flow cytometric plots (a) and cumulative data (b) illustrating CD69 expression on CD4$^+$ T cells in the peripheral blood of healthy controls (HC, n=39), pemphigus (n=19) and SLE (n=23) patients. **(c)** Correlation between the frequency of CD19^{hi} B cells and CD69 expression on CD4$^+$ T cells in the peripheral blood of SLE patients (n=22). Each symbol reflected one sample. Each bar indicated as mean ± S.E.M. **: P < 0.01; ***: P < 0.001.

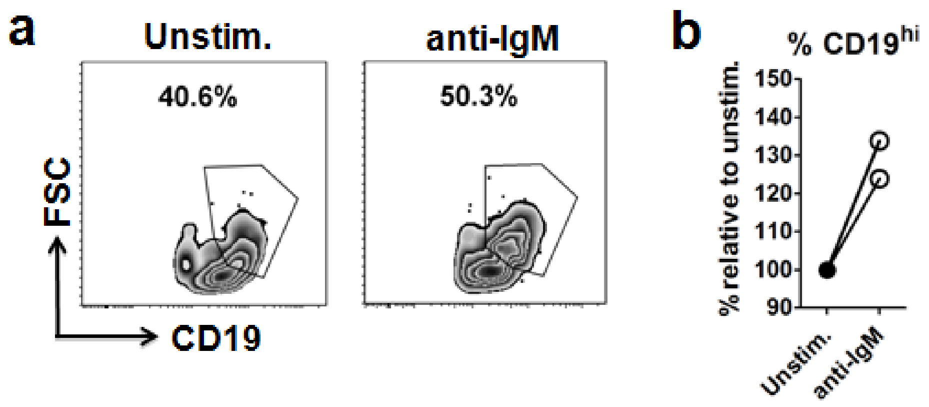

Figure S2. More CD19^{hi} B cells were generated upon anti-IgM stimulation. Freshly isolated B cells were co-cultured with autologous CD4^{+} T cells, and stimulated with or without anti-IgM (Goat F(ab')^{2} anti-Human IgM, Fc5μ fragment specific, Unconjugated; Jackson, USA) *in vitro*. After a 8-day incubation, the cells were subjected to flow cytometry analysis. **(a)** Representative of CD19^{hi} B cells after stimulation with or without anti-IgM. **(b)** Percentage of the CD19^{hi} B-cell frequency relative to the cells unstimulated with anti-IgM. Unstim., Unstimulated.

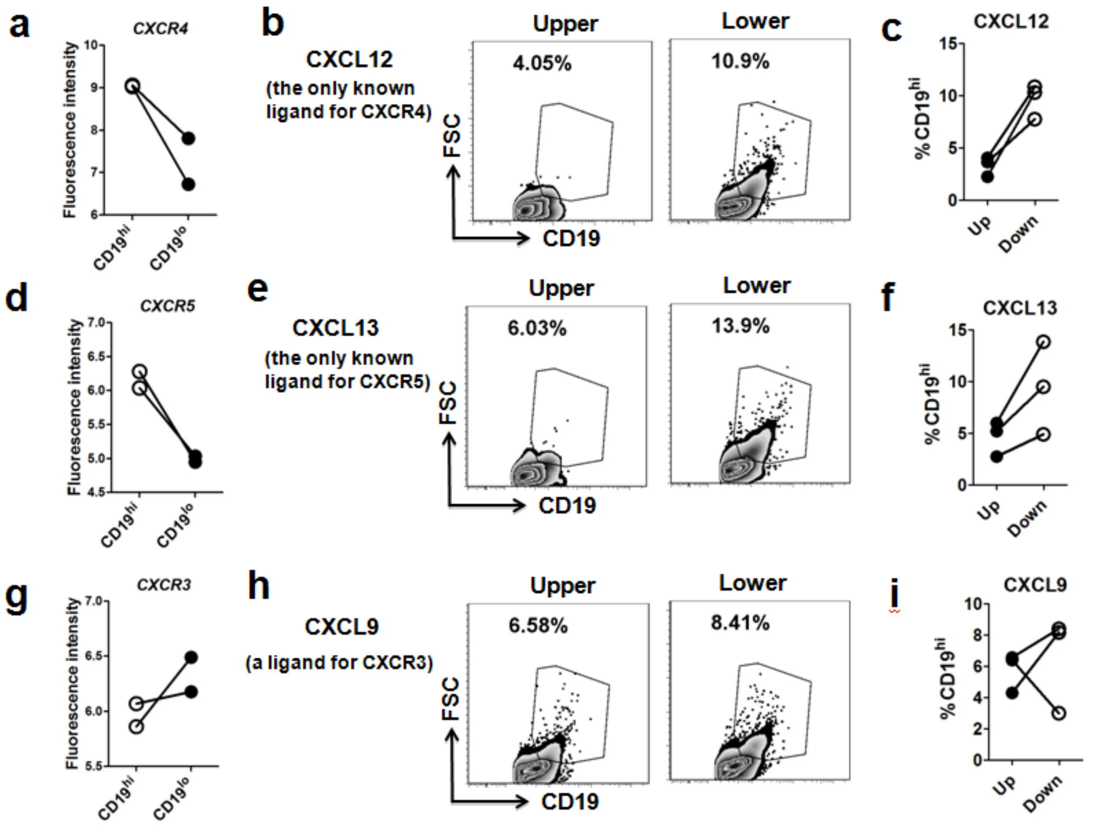

Figure S3. CD19^{hi} B cells exhibited a high migration activity in response to CXCR4 and CXCR5 stimulation, but not to CXCR3 stimulation. (a, d and g) Comparison of gene expression of *CXCR4* (a), *CXCR5* (d) and *CXCR3* (g) between CD19^{hi} and CD19^{lo} B cells from microarray dataset. **(b, c, e, f, h and i)** Transwell migration assay revealed that CD19^{hi} B cells exhibit a high migration activity in response to CXCR4 and CXCR5 stimulation. Peripheral isolated CD4$^+$ T cells and B cells were placed into the upper chambers of the transwell inserts (8-μm pore size; Corning, USA). Mediums containing recombinant human MIG (CXCL9, a ligand for CXCR3), SDF-1α (CXCL12, the only known ligand for CXCR4) or BCA-1 (CXCL13, the only known ligand for CXCR5) were loaded on the lower chambers of

24-well transwell plates. After a 24-hour incubation, the cells in both upper and lower chambers were subjected to flow cytometry analysis.
